# Loganin Ameliorates Painful Diabetic Neuropathy by Modulating Oxidative Stress, Inflammation and Insulin Sensitivity in Streptozotocin-Nicotinamide-Induced Diabetic Rats

**DOI:** 10.3390/cells10102688

**Published:** 2021-10-08

**Authors:** Yu-Chi Cheng, Yu-Min Chiu, Zen-Kong Dai, Bin-Nan Wu

**Affiliations:** 1 Drug Development and Value Creation Research Center, Department of Pharmacology, Graduate Institute of Medicine, College of Medicine, Kaohsiung Medical University, Kaohsiung 807, Taiwan; u9251055@gmail.com (Y.-C.C.); jsh70296@gmail.com (Y.-M.C.); 2 Department of Pediatrics, School of Medicine, College of Medicine, Kaohsiung Medical University, Kaohsiung 807, Taiwan; 3 Department of Pediatrics, Division of Pediatric Cardiology and Pulmonology, Kaohsiung Medical University Hospital, Kaohsiung 807, Taiwan; 4 Department of Medical Research, Kaohsiung Medical University Hospital, Kaohsiung 807, Taiwan

**Keywords:** loganin, painful diabetic neuropathy, oxidative stress, inflammation, insulin resistance

## Abstract

Loganin is an iridoid glycoside with antioxidant, anti-inflammatory, glucose-lowering activities which may address the pathological mechanisms of painful diabetic neuropathy (PDN) related to inflammation, oxidative stress, and hyperglycemia. This study investigated the underlying mechanisms of action of loganin on PDN. The in vivo model of PDN was established by streptozotocin-nicotinamide (STZ-NA) induction in Sprague Dawley (SD) rats. Subsequently, loganin (5 mg/kg) was administered by daily intraperitoneal injection. High-glucose stimulated human SH-SY5Y cells co-incubated with loganin were used to mimic the in vitro model of PDN. Loganin improved PDN rats’ associated pain behaviors (allodynia and hyperalgesia), insulin resistance index (HOMA-IR), and serum levels of superoxide dismutase (SOD), catalase and glutathione. Loganin also reduced pain-associated channel protein Ca*_V_*3.2 and calcitonin gene-related peptide (CGRP) in the surficial spinal dorsal horn of PDN rats. Loganin inhibited oxidative stress and NF-κB activation and decreased the levels of mRNA and protein of proinflammatory factors IL-1β and TNF-α. Moreover, loganin attenuated insulin resistance by modulating the JNK-IRS-1 (insulin receptor substrate-1)-Akt-GSK3β signaling pathway in PDN rats. These results suggested that loganin improved PDN-mediated pain behaviors by inhibiting oxidative stress-provoked inflammation in the spinal cord, resulting in improved neuropathic pain.

## 1. Introduction

Diabetic neuropathy is a common complication of type 2 diabetes mellitus (T2DM), which develops in at least 50% of diabetic patients and usually affects the sensory, motor, and autonomic nervous systems [1,2]. Painful diabetic neuropathy (PDN) is defined as pain resulting from abnormalities in the peripheral somatosensory system in people with diabetes. It is related to abnormal sensory signs of small-fiber and large-fiber neuropathy [3,4]. Most patients develop small-fiber neuropathy in the early stage of diabetic neuropathy or when diagnosed with prediabetes. Up to 25% of patients with diabetic neuropathy will experience neuropathic pain, mainly hyperalgesia or allodynia [5].

T2DM is characterized by hyperglycemia, insulin resistance, and relative insulin deficiency [6]. The pathological mechanism of PDN is related to inflammation caused by persistent hyperglycemia to produce reactive oxygen species [7]. Oxidative damage to the peripheral nerves causes hyperexcitability in the afferent nociceptors and central neurons, producing spontaneous impulses in axons and dorsal root ganglia [8]. Evidence supports the generation of advanced glycation end product, mitochondrial dysfunction and activation of nuclear factor-κB (NF-κB), leading to oxidative stress, in the development of diabetic neuropathy [9,10]. During hyperglycemia, proinflammatory cytokines, including tumor necrosis factor-α (TNF-α) and interleukin-1β (IL-β), elevate and cause nerve cell damage [11]. 

Insulin resistance in neurons leads to peripheral and central nervous system damage and dysfunction. It modulates insulin signaling, affecting downstream phosphatidylinositol 3-kinase (PI3K)/Akt signaling that mediates various downstream biological insulin responses, including cell survival and glucose metabolism [12]. Neuropathic pain is associated with the downregulation of insulin receptors and insulin resistance [13]. Conversely, intensive glycemic control is associated with increased nerve regeneration and improved pain in patients with PDN [14]. A 6-year follow-up study by Cho et al. found that diabetic neuropathy is affected by previous insulin resistance despite regular glycemic control [15]. Accordingly, blood glucose and insulin resistance must be controlled to maintain normal sensory nerve functions in diabetic neuropathy.

Loganin, an iridoid glycoside isolated from *Cornus officinalis*, has exhibited various biological properties, including anti-inflammatory, antioxidant, and anti-apoptotic effects [16,17]. Mo et al. showed the antidiabetic effect of loganin inhibition of FOXO1 nuclear translocation via the PI3K/Akt signaling pathway in pancreatic β-cells [18]. Loganin alleviates depression and anxiety behaviors and diabetes by reducing blood glucose and proinflammatory cytokines [19]. Our previous studies revealed that loganin prevents neuropathic pain by reducing the activation of NF-κB mediated by TNF-α and IL-1β in a chronic constriction injury rat model [20]. We also showed that loganin reduces high glucose-induced Schwann cell pyroptosis by inhibiting ROS generation and NLRP3 inflammasome activation [21]. However, the molecular mechanisms of loganin’s effects on PDN remain unknown. This study explored the mechanisms of the antioxidant and anti-inflammatory effects of loganin on PDN and associated insulin resistance. 

## 2. Materials and Methods

### 2.1. Materials

A regular chow diet (maintenance diet 1320; Altromin, Lage, Germany) was provided by the Center for Laboratory Animals of Kaohsiung Medical University. A high-fat diet (D12492) was obtained from Research Diets Inc. (New Brunswick, NJ, USA).

The following items were purchased from the cited commercial sources: streptozotocin (STZ, S0130), nicotinamide (NA, N3376), all-trans-retinoic acid (R2625), *N*-acetylcysteine (NAC, A9165), 2’,7’-dichlorodihydrofluorescein diacetate (H_2_DCFDA, D6883), TriPure isolation reagents, FITC-conjugated isolectin B4 (L2895), Fluoroshield with DAPI (F6057) and mouse anti-β actin (A5441) from Sigma-Aldrich (St. Louis, MO, USA), glucometer (Rightest, GS700) and glucose test strips from Bionime (San Diego, CA, USA), loganin (19997) and superoxide dismutase activity kits (706002) from Cayman Chemical (Ann Arbor, MI, USA), high-capacity cDNA reverse transcription kits (4368814) from Applied Biosystems (Carlsbad, CA, USA), ChamQ Universal SYBR qPCR Master Mix (^#^Q711) from Vazyme (Nanjing, China), rat tumor necrosis factor α (TNF-α, ^#^RTA00) and interleukin-1β (IL-1β, ^#^RLB00) enzyme-linked immunosorbent assay (ELISA) kits from R&D Systems (Minneapolis, MN, USA), rat insulin ELISA kits (10-1250-01) from Mercodia AB (Uppsala, Sweden), catalase (E-BC-K031-M) and reduced glutathione (E-BC-K030-M) activity assay kits from Elabscience Biotech (Wuhan, China), Eagle Minimum Essential Medium (EMEM), Ham’s F12 medium, T-PER™ tissue protein extraction reagent, M-PER™ mammalian protein extraction reagent, and Pierce™ BCA protein assay kits from Thermo Fisher Scientific (Waltham, MA, USA), quinazoline (QNZ, S4902) from Selleckchem (Houston, TX, USA), anti-phospho-NF-κB (ser^536^, ^#^3033), anti-NF-κB (^#^6956), anti-CGRP (ab47027), anti-phospho-JNK (Thr^183^/Tyr^185^, ^#^4668), and anti-JNK2 (^#^9258) antibodies from Cell Signaling (Danvers, MA, USA), anti-IL-1β (ab9722), anti-phospho-GSK3β (Ser^9^, ^#^9323), anti-GSK3β (^#^12456), anti-phospho-Akt (ser^473^, ^#^4060), and anti-Akt (^#^9272) antibodies from Abcam (Cambridge, MA, USA), anti-TNF-α antibody (NBP-19532) from Novus Biologicals (Littleton, CO, USA), anti-insulin receptor substrate-1 (anti-IRS1, AP-0552) from Proteintech (Wuhan, China), anti-phospho-IRS1 (ser307, 17509-1-AP) from ABclonal (Woburn, MA, USA) and anti-Ca*_V_*3.2 antibody (ACC-025) from Alomone Labs (Jerusalem, Israel).

### 2.2. Animals

Male 6-week-old Sprague Dawley (SD) rats were purchased from BioLASCO Taiwan Co., Ltd. (Taipei, Taiwan) and used in this study at 8 weeks of age. All experimental animal protocols were reviewed and approved by the Institutional Animal Care and Use Committee of Kaohsiung Medical University of Taiwan (IACUC No. 108121) and conducted under the Guide for the Care and Use of Laboratory Animals. The animals were kept under specific pathogen-free (SPF) conditions in polypropylene cages at 22 ± 1 °C and 60 ± 10% relative humidity with a 12 h light/12 h dark cycle, and had free access to water and feed.

### 2.3. Painful Diabetic Neuropathy Rat Model and Experimental Design

After one week of acclimatization, 6-week-old rats were free to access food and water. Briefly, 40 rats were divided into the control and diabetic groups, and the control groups (*n* = 20) were fed a regular diet (maintenance diet 1320; 11% kcal from fat; 65% kcal from carbohydrate; 24% kcal from protein). The diabetic groups (*n* = 20) received a high-fat diet containing 25% soybean oil (D12492: 60% kcal from fat; 20% kcal from carbohydrate; 20% kcal from protein) for the experimental duration. After one week, overnight fasted rats (8-week-old) in the diabetes group underwent induced diabetes by intraperitoneal injection of STZ (55 mg/kg) followed by NA (200 mg/kg) within 15 min for 2 days, as previously described [22]. STZ was dissolved in 0.1 M citrate buffer (pH 4.5). Nicotinamide was dissolved in normal saline. The rats in the control group received the same volume of normal saline and 0.1 M citrate buffer intraperitoneally. The fasting blood glucose level was measured three days after induction using a blood glucose meter and blood glucose test strips from tail vein blood samples. Rats with blood glucose above 200 mg/dl were considered as diabetic. PDN was defined as both thermal withdrawal latency (TWL) and paw withdrawal threshold (PWT) of ≤85% of the base value for this study [23]. After the PDN model was well established, animals were assigned to four subgroups of 10 rats: control group, PDN group, control + loganin group, and PDN + loganin group. Loganin was dissolved in saline and administered at a daily intraperitoneal dose of 5 mg/kg for 4 weeks. The dose of loganin was based on our previous study of chronic constriction injury and a study of diabetic neuropathy by Jiang et al. [20,24]. Body weight and fasting blood glucose levels were measured weekly in all groups. All animals were anesthetized with isoflurane before sacrifice. Plasma and the lumbar spinal cord (L4–L6) were collected from all rats and stored at a −80 °C freezer until analysis.

### 2.4. SH-SY5Y Cell Culture and Treatment

Undifferentiated SH-SY5Y cells (ATCC, CRL-2266) were cultured in a 1:1 mixture of EMEM (5.6 mM d-glucose)/F12 (10 mM d-glucose) containing an average of 7.8 mM d-glucose and supplemented with 10% fetal bovine serum (FBS), 1% GlutaMAX™, 100 U/mL penicillin, and 100 μg/mL streptomycin at 37 °C in a 5% CO_2_ humidified atmosphere and split at 80–90% confluence. In this study, all cells were fully differentiated to a mature neuronal-like state. Cell differentiation was induced by adding 10 µM all-trans-retinoic acid in 1% FBS medium for five days. At 70% confluence, cells were synchronized by serum starvation for 4 h. SH-SY5Y cells were pretreated with 10 μM loganin or 10 nM quinazoline (QNZ; as a NF-κB inhibitor) or 1 mM *N*-acetylcysteine (NAC; as a ROS scavenger) for 2 h and then treated with glucose (HG; 40 mM glucose) for 24 h. Cell experiments were performed within 10 passages.

### 2.5. Pain Sensitivity Assay

Behavioral tests were performed the day before STZ-NA induction, upon starting loganin treatment, and following weeks 1, 2, 3 and 4. Experiments were performed 2 h after loganin administration.

For PWT assessment, animals were placed in individual plastic boxes and allowed to adapt. The plantar surface of each hind paw was perpendicularly stimulated using a dynamic plantar aesthesiometer (DPA; Ugo Basile, Comerio Varese, Italy). A Von Frey-type 0.5 mm rigid filament exerts an increasing force (2.5 g/s with a cutoff time of 20 s) until the animal twitches its paw. A quick withdrawal of the hind paw on stimulus was recorded as a positive response. Each rat was repeated 5 times at 10 min intervals, and the average was used as the PWT.

The TWL was evaluated by a Plantar test (Hargreaves Apparatus) (Ugo Basile, Comerio Varese, Italy). Each rat was placed on a glass plate and allowed to acclimatize. Heat stimulation was applied at infrared intensity (IR) 50. The reaction time of the rats from the beginning of radiant heat measurement until the rat removed its paw was automatically recorded as the TWL, with a cutoff of 20 s to prevent tissue damage. The average was taken of 5 latencies for each hind paw measured at 10 min intervals.

### 2.6. Determination of Insulin and Calculation of HOMA-IR

On the test day, rats were fasted for at least 8 h after injection of loganin. The fasting plasma insulin levels were determined using a rat insulin ELISA kit according to the manufacturer’s instructions. Fasting plasma glucose levels were measured by a glucometer according to the supplier’s instructions. Insulin resistance was determined by homeostatic model assessment of insulin resistance (HOMA-IR) using the following Equation (1) [25]:

(1) $$HOMA-IR=\frac{Fasting\;insulin\;\left(mIU/L\right)\times Fasting\;glucose\;\left(mg/dL\right)}{405}$$

### 2.7. ELISA for Cytokines and Oxidative Stress Biomarkers

Blood samples were collected from the tail vein before the rats were sacrificed. After that, circulating cytokines and oxidative stress biomarkers were measured. The plasma levels of inflammatory cytokines including IL-1β and TNF-α were determined using ELISA kits. Superoxide dismutase (SOD), catalase (CAT), and reduced glutathione (GSH) were measured with commercial kits to test oxidative stress using a spectrophotometer (Synergy^TM^ H1, BioTek, Winooski, VT, USA). All kits were used according to the manufacturer’s instructions.

### 2.8. Western Blot Analysis

Protein was extracted from the spinal cord segments using T-PER containing EDTA-free protease inhibitor cocktail and PhosSTOP phosphatase inhibitor. SH-SY5Y cells were seeded in 10 cm dishes (1 × 10^6^ cells) with lysis by M-PER containing proteinase inhibitors after treatment. The lysates were separated by SDS-PAGE (7.5–12%), transferred to PVDF membrane in blocking buffer (Tris-buffered saline with 0.1% Tween-20 buffer containing 3% bovine serum albumin) and incubated with primary antibodies at 4 °C overnight. Primary antibody anti-phospho (ser^536^) NF-κB, anti-NF-κB, anti-TNF-α, anti-IL-1β, anti-phospho (ser^307^)-IRS-1, anti-IRS-1, anti-phospho (thr^183^/tyr^185^)-JNK, anti-JNK2, anti-phospho (ser^9^)-GSK-3β, anti-GSK-3β, anti-phospho (ser^473^)-Akt, and anti-Akt antibodies were diluted and detected using appropriate peroxidase-conjugated secondary antibodies. β-actin was used as an internal control to ensure equal loading. Signals were detected with a Chemiluminescent HRP Substrate reagent and quantized by densitometry with Image-J software (NIH, Bethesda, MD, USA).

### 2.9. Immunofluorescence

The L4–L6 spinal cord regions were isolated and fixed in 10% neutral buffered formalin for 2 h at 4 °C. Subsequently, the spinal cord samples were equilibrated in a 30% sucrose solution at 4 °C overnight. The tissues were embedded in optimal cutting temperature (OCT) compound and flash-frozen in liquid nitrogen. Tissue sections were sliced at a thickness of 12 µm by a cryostat (Leica CM1800; Heidelberg, Germany). SH-SY5Y cells were seeded on glass coverslips and fixed with 10% neutral buffered formalin after treatment, washed twice in phosphate-buffered saline (PBS) and permeabilized with 0.1% Triton X-100 in PBS. Samples were blocked with 3% bovine serum albumin in PBS and incubated with anti-CGRP, anti-TNF-α, anti-IL-1β and anti-phospho-NF-κB primary antibodies for 16 h at 4 °C. Next, the slides were incubated with Alexa Fluor 488 goat anti-rabbit IgG or FITC-conjugated IB4 for 1 h at room temperature. Slides were mounted with Fluoroshield with DAPI. Images were acquired by a Leica DMi8 inverted light microscope with Leica Application Suite X software (Version 3.0.3) (Leica, Wetzlar, Germany) to process the image. The mean gray values of images or phosphor-NF-κB puncta were measured and quantified in >10 randomly selected images using Image J software.

### 2.10. RNA Extraction, cDNA Synthesis and Quantitative Real-Time PCR (qRT-PCR)

Total RNA was extracted from spinal cord samples using TriPure reagent. Total RNA (1 μg) was reverse transcribed into cDNA using the high-capacity cDNA reverse transcription kit. qRT-PCR was performed with the StepOnePlus Real-time PCR system (Applied Biosystems) using 2× ChamQ Universal SYBR qPCR Master Mix. PCR reactions were performed under the following conditions: 10 min at 95 °C and 40 cycles of the one-step thermal cycling of 3 s at 95 °C and 30 s at 60 °C. The primer sequences used were *TNF-α* forward, 5′-CTC AAG CCC TGG TAT GAG CC-3′ and reverse, 5′-GGC TGG GTA GAG AAC GGA TG-3′; *IL-1β* forward, 5′-AAA TGC CTC GTG CTG TCT GA-3′ and reverse, 5′-AGG CCA CAG GGA TTT TGT CG-3′ and *β-actin* forward, 5′-GAC CCA GAT CAT GTT TGA GAC C-3′ and reverse, 5′-AGG CAT ACA GGG ACA ACA CA-3′. The relative gene expression levels of *TNF-α* and *IL-1β* were analyzed by the 2^−ΔΔCt^ method and normalized to β-actin. All reactions were performed in triplicate.

### 2.11. Measurement of Intracellular ROS

Intracellular ROS levels were detected using a H_2_DCFDA dye method. Differentiated SH-SY5Y cells were seeded in 24 well plates (2 × 10^4^ cells/well) and 10 μM dye was added for 30 min at 37 °C in a CO_2_ incubator before treatment. From the DCF fluorescence, we measured intracellular ROS with a Leica DMi8 inverted light microscope with Leica Application Suite X software to process the image. The mean gray values of images were measured and quantified in >10 randomly selected images using Image J software.

### 2.12. Cell Viability Assays

Differentiated SH-SY5Y cells were seeded into 96-well plates at a density of 2 × 10^3^ cells/well and incubated under the different experimental conditions. Cell viabilities were detected using a Cell Counting Kit-8 (CCK-8, Biotools, Taipei, Taiwan) according to the manufacturer’s instructions. After treatment, the medium was refreshed and 10 μL of the CCK-8 solution was added to each well. After incubation for 2 h at 37 °C, the value of optical absorbance at 450 nm (with 650 nm as reference) was determined using a microplate reader (Synergy^TM^ H1, BioTek, Winooski, VT, USA).

### 2.13. Statistical Analysis

Statistical analyses were performed using GraphPad Prism 7.0 software. Differences in body weight, fasting blood glucose levels, PWT and TWL were analyzed by a two-way analysis of variance (ANOVA) followed by Bonferroni’s post hoc tests. All other data were analyzed using one-way ANOVA followed by a Tukey–Kramer post hoc test. Data are represented as the mean ± standard error of the mean (SEM) with the statistical significance level set at *p* < 0.05.

## 3. Results

### 3.1. Loganin Ameliorated Hyperglycemia, Pain Behaviors and Insulin Resistance in STZ-NA Injected Rats

**Figure 1:**
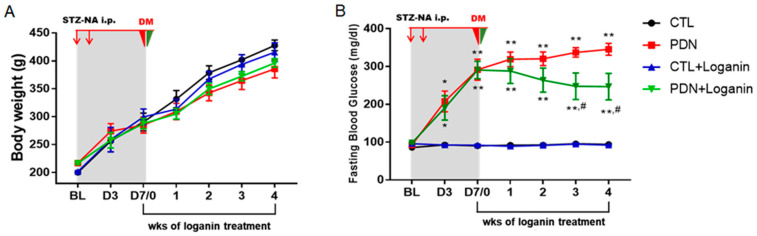

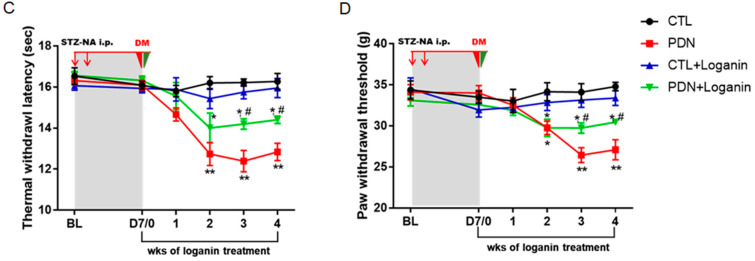
Effects of loganin on body weight, fasting blood glucose, thermal hyperalgesia and mechanical allodynia in STZ-NA-induced diabetic rats. (**A**) Body weight and (**B**) fasting blood glucose were measured on the day of STZ/NA induction (BL), days 3 and 7 after STZ/NA induction, and weeks 1, 2, 3 and 4 after loganin treatment. Pain behaviors were measured by estimating (**C**) thermal withdrawal latency and (**D**) paw withdrawal thresholds on days 0 and 7 after STZ/NA induction and weeks 1, 2, 3 and 4 after loganin treatment. All data are presented as mean ± SEM. * *p* < 0.05 vs. CTL group, ** *p* < 0.01 vs. CTL group; ^#^ *p* < 0.05 vs. PDN group, *n* = 8. STZ: streptozotocin, NA: nicotinamide, PDN: painful diabetic neuropathy, BL: baseline, CTL: control.

As shown in Figure 1A, after STZ-NA injection there was no significant change in body weight between the groups weekly. After seven days of STZ-NA induction, the fasting blood glucose levels were significantly above 200 mg/dL and daily intraperitoneal injection of loganin (5 mg/kg) was started. After three weeks of treatment with loganin, the fasting blood glucose levels of PDN rats were significantly reduced but still significantly higher than in the control group (Figure 1B).

Two pain behaviors (TWL and PWT) were assessed to verify the pain conditions with and without loganin in T2DM rats induced by STZ-NA. Two weeks after STZ-NA injection, the pain behaviors of TWL and PWT were significantly reduced. Three weeks after the injection of loganin, the pain threshold of PDN rats increased, though it was still lower than the control group (Figure 1C,D).

Next, we estimated the protective effects of loganin on insulin resistance. HOMA-IR is calculated to evaluate insulin resistance [26]. The fasting blood glucose, fasting plasma insulin, and computed HOMA-IR score were detected in the 4th week (Table 1). Of note, even if there were no significant changes in fasting plasma insulin levels, the HOMA-IR score of PDN rats was significantly higher than that of the control group. It was reduced after four weeks of loganin treatment, although still higher than the control group.

Collectively, after two weeks of STZ-NA induction, rats developed PDN, although there were no significant changes in body weight and fasting insulin. After daily loganin treatment for three weeks, the blood sugar, pain behaviors and insulin resistance of PDN rats were all improved.

### 3.2. Loganin Decreased Ca_V_3.2 T-type Calcium Channels, CGRP Expression and Glial Activation in the Spinal Dorsal Horn of PDN Rats

**Figure 2:**
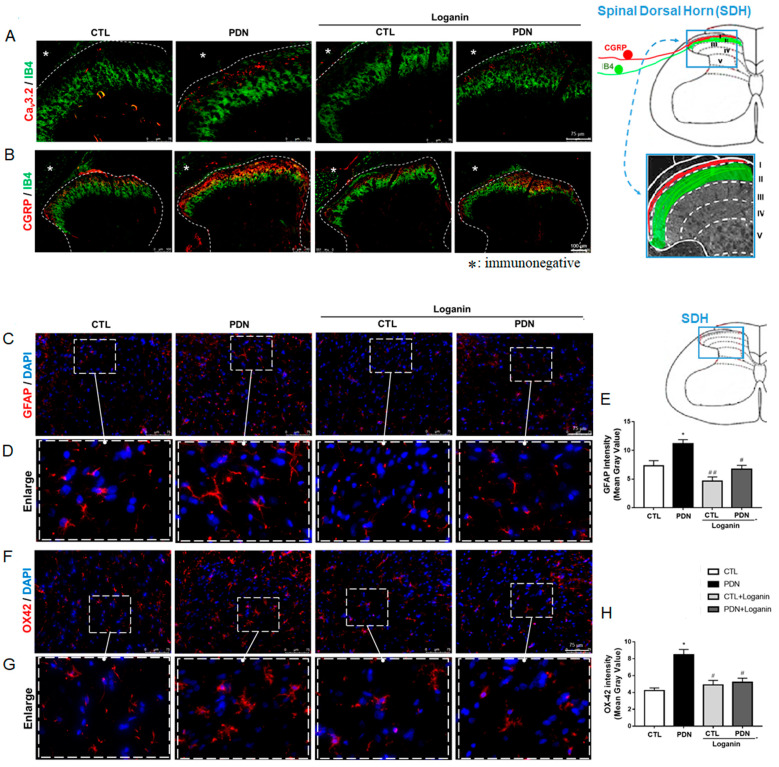
Effects of loganin on Ca*_v_*3.2, CGRP expression and glial activation in the spinal dorsal horn in PDN rats. Representative immunofluorescence images: (**A**) Ca*_V_*3.2 (red) with presynaptic markers IB4 (green). Scale bar = 75 µm. (**B**) CGRP (red, neuropeptide) with IB4 (green, labeled afferents terminate in inner lamina II). Scale bar = 100 µm. (**C**) GFAP (red, astrocyte marker) with DAPI (blue, nuclei). Scale bar = 75 µm. (**D**) The inset below shows an enlarged image. (**F**) OX42 (red, microglial marker) with DAPI. Scale bar = 75 µm. (**G**) The inset below shows higher magnification. (**E**,**H**) mean gray values of GFAP and OX42 staining in spinal dorsal horn (*n* = 10 sections from 4 rats, * *p* < 0.05 vs. CTL group; ^#^ *p* < 0.05 vs. PDN group, ^##^ *p* < 0.01 vs. PDN group). CTL: control, PDN: painful diabetic neuropathy, GFAP: glial fibrillary acidic protein, CGRP: calcitonin gene-related peptide, IB4: isolectin B4.

To investigate the distribution of pain-associated proteins, Ca*_V_*3.2 T channel subtypes and CGRP in the spinal dorsal horn (SDH) and the impact of loganin, immunofluorescence was employed using isolectin B4 (IB4) as a presynaptic nerve terminal marker in lamina II of the SDH [27]. We found that Ca*_V_*3.2 fluorescence increased in the superficial SDH in PDN rats and reduced in loganin-treated rats (Figure 2A). We also examined the distribution of CGRP. Immunofluorescent imaging was focused on lamina I–II as indicated in the upper right of Figure 2. The images showed stronger CGRP fluorescence (Figure 2B) in lamina I–II of the SDH in PDN rats than in loganin-treated rats.

We investigated the regulatory effect of loganin on astrocyte and microglia activation in the PDN rats. GFAP and OX-42 (CD11b) were used as astrocyte and microglia markers, respectively. The GFAP (Figure 2C,D) and OX-42 (Figure 2F,G) immunoreactive areas indicating activated astrocyte and microglia were markedly larger in the SDH sections from PDN rats when compared with other groups. The mean gray values of staining were measured by Image J software. These quantitative data showed that the activation of astrocytes and microglia was decreased after loganin treatment in PDN rats (Figure 2E,H).

Based on these results, loganin might decrease Ca*_V_*3.2 T-type calcium channels and neurotransmitter CGRP distribution in superficial SDH and reduce glial cell activation in PDN rats, related to the improvement of neuropathic pain in T2DM rats.

### 3.3. Loganin Suppressed Oxidative Stress and Proinflammatory Factors in the Serum of PDN Rats

**Figure 3:**
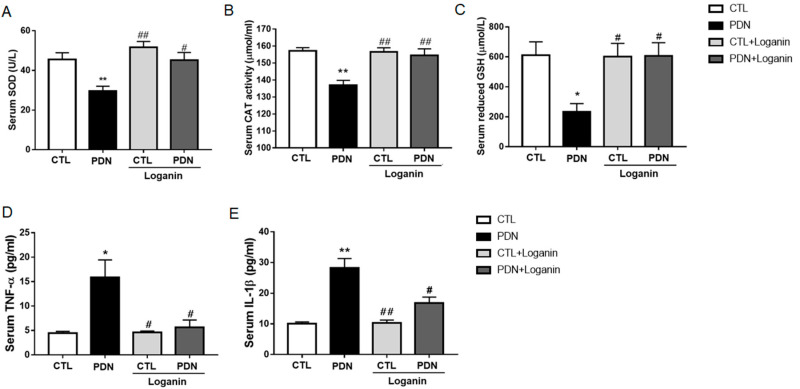
Effects of loganin on antioxidant enzymatic activities and proinflammatory factor release in the serum of PDN rats. The enzymatic activities of (**A**) SOD, (**B**) CAT and (**C**) reduced GSH, and the serum levels of (**D**) TNF-α and (**E**) IL-1β were measured by ELISA. Data are expressed as the mean ± SEM (*n* = 8). * *p* < 0.05 vs. CTL group, ** *p* < 0.01 vs. CTL group; ^#^ *p* < 0.05 vs. PDN group, ^##^ *p* < 0.01 vs. PDN group. CTL: control, PDN: painful diabetic neuropathy, SOD: superoxide dismutase, CAT: catalase, GSH: glutathione, TNF-α: tumor necrosis factor-α, IL-1β: interleukin-1β.

To clarify the antioxidant and inflammatory effects of loganin on PDN rats, we tested the activity of antioxidant enzymes and the content of proinflammatory factors in PDN rat serum. Compared with the control group, the activity of antioxidant enzymes in PDN rats, including superoxide dismutase (SOD), catalase (CAT), and glutathione peroxidase (GSH), were decreased and then recovered after loganin for four weeks (Figure 3A–C). We also analyzed the serum levels of proinflammatory factors (TNF-α, IL-1β). Our results showed that loganin reduced TNF-α and IL-1β in the serum of PDN rats (Figure 3D,E).

### 3.4. Loganin Diminished Inflammatory Mediators by Inhibiting NF-κB Activation in the Spinal Cord Tissue of PDN Rats

**Figure 4:**
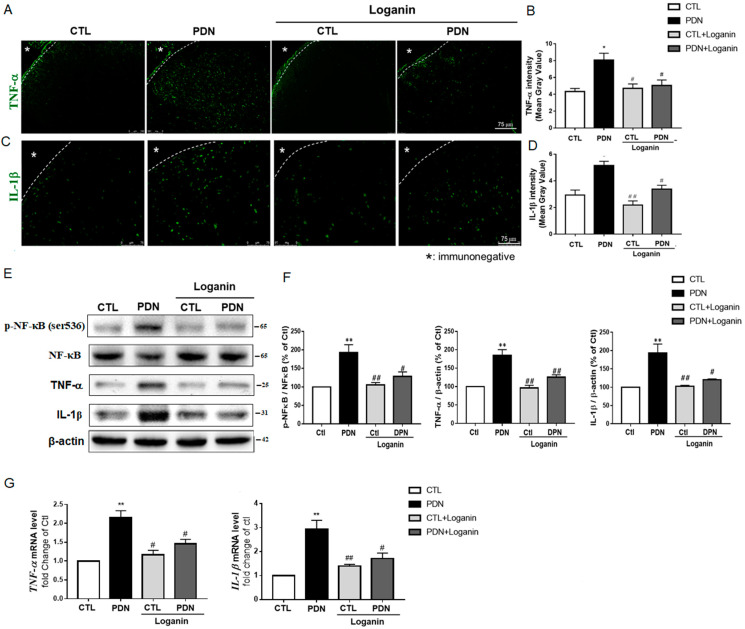
Effects of loganin on the production of inflammatory mediators and the activation of NF-κB in rat spinal cord tissue. Immunofluorescence staining revealed (**A**) TNF-α (green) and (**C**) IL-1β (green). Scale bar =75 μm. (**B**) and (**D**) represent the mean gray value, respectively (*n* = 10 sections from 4 rats, * *p* < 0.05 vs. CTL group; ^#^ *p* < 0.05 vs. PDN group, ^##^ *p* < 0.01 vs. PDN group). Western blot images showed (**E**) the expression of p-NF-κB (ser^536^), NF-κB, TNF-α and IL-1β proteins and (**F**) phosphorylation of NF-κB and fold changes of TNF-α, and IL-1β proteins. β-actin was used as a loading control. Gene expression by RT-qPCR of (**G**) *TNF-α* and *IL-1β* is shown after normalization to β-actin as a fold change over CTL (*n* = 4). * *p* < 0.05 vs. Ctl group, ** *p* < 0.01 vs. CTL group; ^#^ *p* < 0.05 vs. PDN group, ^##^ *p* < 0.01 vs. PDN group. Data expressed as mean ± SEM. CTL: control, PDN: painful diabetic neuropathy, NF-κB: nuclear factor-κB, TNF-α: tumor necrosis factor-α, IL-1β: interleukin-1β.

To further verify the anti-inflammatory effects and possible mechanism of loganin, the L4-5 lumbar segments of the spinal cord were removed for study. Immunofluorescence staining images displayed TNF-α and IL-1β distributed in the spinal dorsal horn region of the PDN group, with decreased expression after loganin treatment (Figure 4A–D). Transcription factor NF-κB is the most studied intracellular pathway related to hyperglycemia, ROS, and oxidative stress. Western blotting data showed significantly decreased NF-κB phosphorylation, TNF-α, and IL-1β protein expression after loganin treatment (Figure 4E,F). In the PDN group, the transcription factor NF-κB was suppressed, and the expression of TNF-α and IL-1β genes was blocked, but there was no significant change in the other groups (Figure 4G). These results revealed that the activation of NF-κB and the gene and protein expression of TNF-α and IL-1β were inhibited after loganin treatment.

### 3.5. Loganin Regulated the JNK-IRS1-AKT-GSK3β Insulin Resistance Pathway in PDN Rat Spinal Cord Tissue

**Figure 5:**
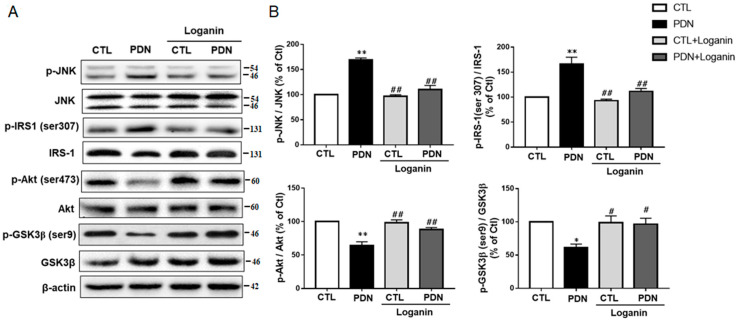
Effects of loganin on the insulin signaling pathway in rat spinal cord tissue. (**A**) Western blot images of relevant proteins: p-JNK (Thr^183^/Tyr^185^), JNK, p-IRS1 (ser^307^), IRS1, p-Akt (ser^473^), Akt, p-GSK3β (ser^9^), and GSK3β, with β-actin used as the quantitative reference. (**B**) Densitometry analysis of the relative phosphorylation of JNK, IRS1 on Ser^307^, Akt on Ser^473^ and GSK3β on Ser^9^. Data are presented as means ± SEM (*n* = 6). * *p* < 0.05, ** *p* < 0.01 vs. CTL group; ^#^ *p* < 0.05, ^##^ *p* < 0.01 vs. PDN group. CTL: control, PDN: painful diabetic neuropathy, IRS1: insulin receptor substrate, GSK3β: glycogen synthase kinase 3β.

As Figure 1D data showed, loganin decreased PDN rats’ HOMA-IR score. We were interested in verifying whether loganin could regulate the JNK signaling pathway and mediate insulin resistance. Western blot data showed that the phosphorylation of JNK in the PDN group increased, which activated the phosphorylation at serine^307^ of insulin receptor substrate 1 (IRS-1). Subsequently, the phosphorylation of AKT was reduced, and the phosphorylation at serine^9^ of GSK3β was also reduced (Figure 5A,B). Altogether, these data indicate that loganin might improve insulin resistance in PDN rats by modulating the JNK-IRS1-AKT-GSK3β signaling pathway.

### 3.6. Loganin Had Antioxidant and Anti-Inflammatory Effects in SH-SY5Y Cells Exposed to High Glucose

**Figure 6:**
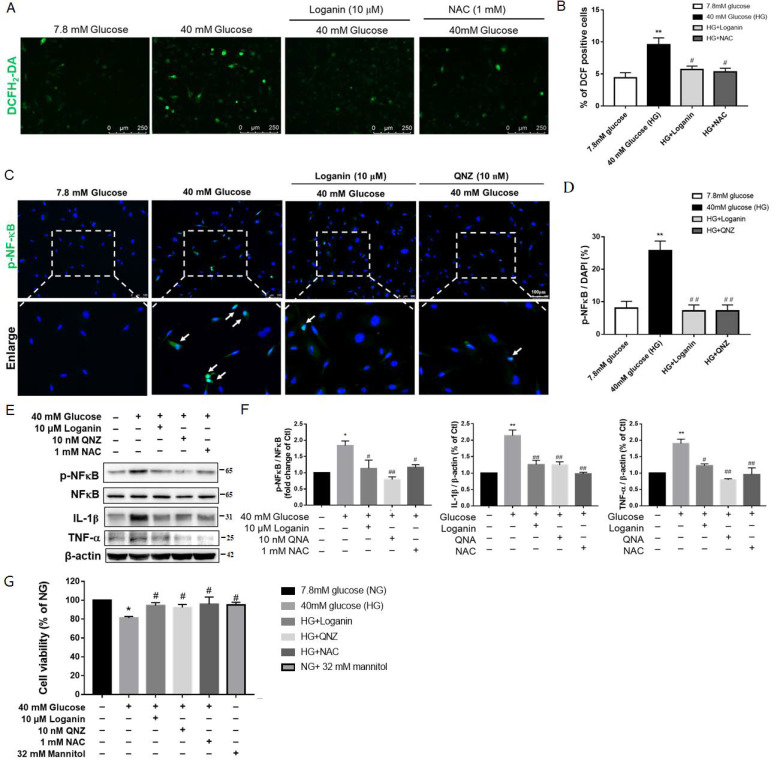
Antioxidant and anti-inflammatory effects of loganin on high-glucose-treated SH-SY5Y cells. (**A**) Images represent intracellular ROS generation with DCF-positive cells in green (scale bar: 250 µm for magnification ×100) and (**B**) reflected as the mean gray value in each view (mean ± SEM, *n* = 10). Immunofluorescence staining revealed (**C**) phosphor-NF-κB (green) and DAPI (blue, nuclei) (scale bar:100 µm for magnification ×200). (**D**) mean gray value, respectively (*n* = 10 images from 4 groups, ** *p* < 0.01 vs. 7.8 mM glucose (NG); ^#^ *p* < 0.05 vs. 40 mM glucose (HG), ^##^ *p* < 0.01 vs. HG). Western blot images show (**E**) the expression of p-NF-κB (Ser^536^), NF-κB, TNF-α and IL-1β proteins and (**F**) phosphorylation of NF-κB and fold changes of TNF-α and IL-1β proteins. β-actin was used as a loading control (*n* = 4). (**G**) Cell viability was measured by cell counting kit-8 (CCK-8). * *p* < 0.05 vs. NG, ** *p* < 0.01 vs. NG; ^#^ *p* < 0.05 vs. HG, ^##^ *p* < 0.01 vs. HG. Data are expressed as mean ± SEM. QNZ: Quinazoline, NAC: *N*-acetylcysteine, ROS: reactive oxygen species, NG: normal glucose, HG: high glucose, NF-κB: nuclear factor-κB, TNF-α: tumor necrosis factor -α, IL-1β: interleukin-1β.

SH-SY5Y is a neuronal cell line commonly used to study diabetic neuropathy [28]. In this study, SH-SY5Y cells exposed to high glucose were used to confirm the mechanisms of the possible protective effects of loganin against oxidative stress and inflammation induced by hyperglycemia to mimic diabetic neuropathy. *N*-Acetylcysteine (NAC) is a commonly used antioxidant. We used it to confirm whether loganin had antioxidative activity against high glucose-induced oxidative stress. SH-SY5Y cells were exposed to high glucose (40 mM) with 1 μM loganin or 1 mM NAC for 24 h and then stained with 10 μM H_2_DCFDA, a membrane-permeable intracellular ROS indicator. The representative fluorescence images revealed that DCF-positive cells were increased markedly by high-glucose stimulation, whereas the presence of loganin prevented this effect (Figure 6A). Quantitatively analyzed data showed that treatment with 1 μM loganin or 1 mM NAC significantly reduced the fluorescence intensity of DCF enhanced by high glucose (Figure 6B).

NF-κB transcription factor is an important mediator of proinflammatory gene production. Quinazoline (QNZ) is a specific NF-κB inhibitor. Loganin suppressed SH-SY5Y cells’ NF-κB translocation to the nucleus after exposure to high glucose. Cells treated with QNZ displayed a similar suppressive effect on NF-κB activation (Figure 6C,D). Western blotting data showed that inhibiting NF-κB phosphorylation also prevented TNF-α and IL-1β protein expression (Figure 6E,F). CCK-8 data showed decreased cell viability in high-glucose-treated SH-SY5Y cells. Cell viability was increased by treatment with loganin, QNZ and NAC. NG plus mannitol was used as an osmotic control (7.8 mM glucose + 32 mM mannitol). The cell viability of SH-SY5Y cells did not show any significant changes under isotonic mannitol conditions (Figure 6G). Collectively, our findings suggest that loganin exerts strong antioxidative and anti-inflammatory activity against high-glucose aggravated cell viability in SH-SY5Y cells.

## 4. Discussion

**Figure 7:**
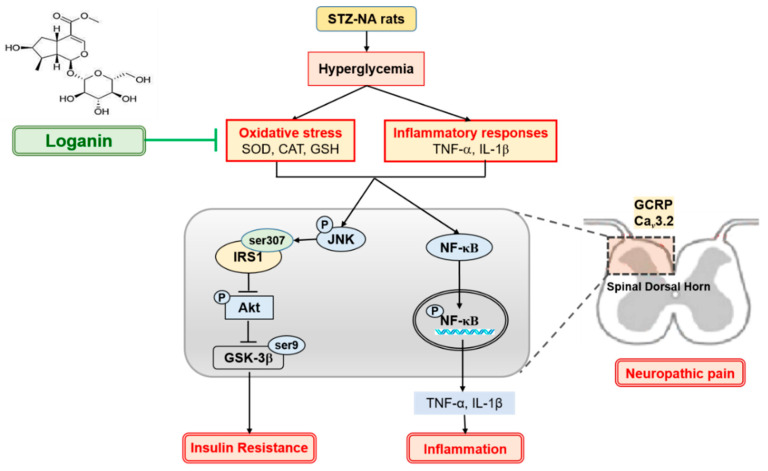
Possible mechanisms of loganin improvement of painful diabetic neuropathy by improving oxidative stress, inflammatory responses and insulin sensitivity in the spinal dorsal horn of type II diabetic rats. STZ: streptozotocin, NA: nicotinamide, SOD: superoxide dismutase, CAT: catalase, GSH: glutathione, TNF-α: tumor necrosis factor-α, IL-1β: interleukin-1β, NF-κB: nuclear factor-κB, IRS1: insulin receptor substrate 1, GSK3β: glycogen synthase kinase 3β, CGRP: calcitonin gene-related peptide.

In the present study, we have shown that nerve injury, including allodynia, hyperalgesia in streptozotocin-nicotinamide-induced T2DM rats, and PDN was exacerbated by oxidative stress and inflammatory responses induced by hyperglycemia and insulin resistance. During diabetes, oxidative stress and proinflammatory cytokines (such as TNF-α and IL-1β) enhance phosphorylation of NF-κB and JNK, causing inflammation and insulin resistance. Loganin relieves inflammation by inhibiting NF-κB phosphorylation, then reducing transcription of TNF-α and IL-1β. Insulin resistance increases since activated JNK induces IRS-1 serine^307^ phosphorylation, inhibiting Akt serine^473^ phosphorylation and subsequent GSK3β serine^9^ phosphorylation. Loganin blunted the phosphorylation of JNK to modulate insulin resistance in PDN rats. Another key to neuropathic pain is that oxidative stress can cause sensory hypersensitivity and increase the expression of Ca*_V_*3.2 channels and CGRP in the superficial dorsal horns (layers I and II). Loganin’s antioxidant effect might improve these abnormalities, as shown in Figure 7.

The pathogenesis of PDN is not fully understood, but there is a consensus that the toxic effects of hyperglycemia play an important role in its development. Hyperglycemia is known to cause disorders of metabolic pathways, which lead to neuronal and axon damage and increased levels of oxidative stress in the nervous system in diabetic neuropathy [3]. Pain and dysesthesia are the most common early symptoms of PDN [29]. In this study, the fasting blood glucose level of PDN rats was higher than that of the control group, and loganin treatment could reduce fasting blood glucose. Although there was no significant difference in fasting serum insulin levels in each group, loganin significantly improved the insulin resistance of PDN rats. Furthermore, PDN rats showed thermal hyperalgesia and mechanical allodynia 14 days after STZ-NA induction that lasted more than two weeks. After daily loganin treatment, the final results revealed that diabetic rats not only had lowered blood glucose and insulin resistance but also improved allodynia and hyperalgesia.

Hyperglycemia is known to aggravate oxidative stress and affect calcium (Ca^2+^) homeostasis. Abnormal neuronal Ca^2+^ homeostasis has been implicated in neuropathic pain and diabetic polyneuropathy [30]. Primary afferent fibers (C and Aδ) that carry nociceptive information and the second-order neurons in the superficial layers (layers I and II) of the spinal dorsal horn are crucial pathways for pain processing [27]. Calcium enters the cytoplasm through voltage-gated calcium channels to trigger calcium-dependent enzyme activation, gene transcription, and presynaptic neurotransmitter release [31]. T-type calcium channels are expressed in the cutaneous mechanoreceptors of Aδ and C fibers both in the initial segment of the axon and in the peripheral terminals in the spinal cord [32]. Feng et al. found that nerve injury elevated functional Cav3.2 channels in the superficial spinal dorsal horn in a neuropathic pain rat model [33]. Recent studies have established that the Ca*_V_*3.2 T-type voltage-gated calcium channel contributes to the hyperexcitability of sensory neurons in PDN animal models [34,35]. Indeed, Ca*_V_*3.2 was upregulated in the surficial layer of the spinal dorsal horn in our PDN group, and daily loganin treatment reversed these circumstances. Moreover, CGRP recognizes small peptidergic neurons in the DRG and the afferents C and Aδ sensory fibers in the spinal cord. It is also thought to contribute to pain transmission and inflammation [36,37]. As predicted, we observed that PDN rats had a widespread CGRP immune response, and the level was limited after treatment with loganin.

Neuropathic pain is caused by multiple factors that lead to increased excitatory conduction in the spinal dorsal horn. This enhanced excitability occurs via a complex four-way communication between primary afferent terminals, dorsal horn neurons, astrocytes and microglia. Microglia and astrocytes help to release various inflammatory mediators, neuromodulators, and growth factors [38]. The proinflammatory cytokines secreted by microglia, such as TNF-α and IL-1β, can induce secondary immune responses in astrocytes to activate the inflammatory pathway of the NF-κB transcription factor, inducing a vicious circle of neuron and glial cell inflammation [39]. As expected, our data showed that astrocytes and microglia were activated in the spinal dorsal horn of PDN rats, the expression of TNF-α and IL-1β was increased, and these effects were reversed by daily loganin administration. As mentioned above, we speculated that loganin might reduce sensory neuron hyperexcitability and glial cell activation by reducing blood glucose and insulin resistance, thereby improving hyperalgesia and allodynia in PDN rats.

NF-κB is the best-known transcription factor related to hyperglycemia, oxidative stress and inflammation, and regulates several gene expressions. Conversely, the gene products regulated by NF-κB can also activate NF-κB (such as IL-1β, TNF-α) [40]. Our animal studies corroborated that loganin inhibited the phosphorylation of NF-κB in the spinal cord of PDN rats. In PDN rats, loganin reduced the serum level of proinflammatory factors (IL-1β and TNF-α), their distribution in the dorsal horn of the spinal cord, and even their mRNA and protein expression. QNZ is a quinazoline derivative that inhibits NF-κB activation, anti-inflammatory and antioxidant activities [41]. After adding QNZ to SH-SY5Y cells treated with high glucose for 24 h, we found that loganin has the same effect as QNZ. It not only reduces the expression of IL-1β and TNF-α but also restores the viability of SH-SY5Y cells treated with high glucose. Furthermore, antioxidant enzyme activities (SOD, CAT and reduced GSH) in the serum of our PDN rats were reduced by treatment with loganin. *N*-Acetylcysteine (NAC) is an exogenous antioxidant that works as a free radical scavenger [42]. We treated SH-SY5Y cells with high glucose and supplemented them with loganin or NAC for 24 h, and the results showed that loganin has antioxidant and anti-inflammatory effects which could prevent neuronal cell damage from high glucose in vivo and in vitro.

Loganin could improve insulin resistance, offering a possible treatment for PDN through its antioxidative stress and anti-inflammatory activities. Of particular note, hyperglycemia-induced oxidative stress also activates JNK, an upstream transcription factor in the insulin resistance signal. The activated JNK further activates IRS-1 [43]. Additionally, both IL-1β and TNF-α simultaneously induce IRS-1 activation [44,45]. Serine phosphorylation targets IRS-1 kinase, which has a negative regulatory effect on IRS-1 function and insulin action. Conversely, tyrosine phosphorylation activates multiple signaling pathways required for insulin action [46]. IRS-1 serine phosphorylation activates downstream Akt. Activation of Akt is a key step to subsequent phosphorylation of GSK3β and activation of glycogen synthase involved in insulin metabolic effects [47]. To explore loganin’s antidiabetic effects, this study investigated the JNK-IRS1-AKT-GSK3β signal pathway. In the spinal cord of PDN rats, activated JNK phosphorylates IRS-1 Ser^307^, which in turn inhibits Akt phosphorylation at Ser^473^ and subsequent GSK3β phosphorylation at Ser^9^, which means that the increase in insulin resistance is due to the inhibition of glucose uptake and glycogen synthesis. Loganin inhibited JNK activation and regulated the IRS-1-AKT-GSK3β signaling pathway by inhibiting oxidative stress, although the regulation of insulin resistance remains to be fully demonstrated.

In conclusion, loganin lowers blood glucose and exerts antioxidant and anti-inflammatory effects, thereby improving insulin resistance and reducing pain behaviors in the rat model of PDN. We suggest that loganin might be a potential agent for treating diabetes and its PDN complications.

## Figures and Tables

**Table 1:** Effects of loganin on fasting blood glucose, fasting plasma insulin and HOMA-IR in PDN rats in week 4. All data are presented as means ± SEM. ** *p* < 0.01 vs. CTL group; ^#^ *p* < 0.05 vs. PDN group, ^##^ *p* < 0.01 vs. PDN group, *n* = 8.

Parameters	Groups			
	CTL	PDN	CTL + Loganin	PDN + Loganin
Fasting blood glucose (mg/dL)	120.5 ± 4.1	428.3 ± 21.0 **	118.3 ± 4.48 ^##^	335.2 ± 41.1 **^,#^
Fasting plasma insulin (mIU/L)	14.5 ± 0.68	13.1 ± 0.41	14.4 ± 0.75	13.2 ± 0.54
HOMA-IR score	3.84 ± 0.17	11.1 ± 0.36 **	4.05 ± 0.23 ^##^	8.82 ± 0.8 **^,#^

CTL: control, PDN: painful diabetic neuropathy, HOMA-IR, homeostasis model assessment-insulin resistance.

## Data Availability

The data that support the findings of this study are available from the corresponding author upon request.
